# Bioprocessing of *Stichococcus bacillaris* strain siva2011

**DOI:** 10.1186/1754-6834-7-62

**Published:** 2014-04-15

**Authors:** Ganapathy Sivakumar, Kwangkook Jeong, Jackson O Lay

**Affiliations:** 1 Arkansas Biosciences Institute and College of Agriculture and Technology, Arkansas State University, PO Box 639, Jonesboro, AR 72401, USA; 2 College of Engineering, Arkansas State University, Jonesboro, AR 72401, USA; 3 Arkansas Statewide Mass Spectrometry Facility, University of Arkansas, Fayetteville, AR 72701, USA

**Keywords:** Algae, Bioreactor, Hydrocarbon, Jet fuel, Triacylglycerol

## Abstract

**Background:**

Globally, the development of a cost-effective long-term renewable energy infrastructure is one of the most challenging problems faced by society today. Microalgae are rich in potential biofuel substrates such as lipids, including triacylglycerols (TAGs). Some of these algae also biosynthesize small molecule hydrocarbons. These hydrocarbons can often be used as liquid fuels, often with more versatility and by a more direct approach than some TAGs. However, the appropriate TAGs, accumulated from microalgae biomass, can be used as substrates for different kinds of renewable liquid fuels such as biodiesel and jet fuel.

**Results:**

This article describes the isolation and identification of a lipid-rich, hydrocarbon-producing alga, *Stichococcus bacillaris* strain siva2011, together with its bioprocessing, hydrocarbon and fatty acid methyl ester (FAME) profiles. The *S. bacillaris* strain siva2011 was scaled-up in an 8 L bioreactor with 0.2% CO_2_. The C16:0, C16:3, C18:1, C18:2 and C18:3 were 112.2, 9.4, 51.3, 74.1 and 69.2 mg/g dry weight (DW), respectively. This new strain produced a significant amount of biomass of 3.79 g/L DW on day 6 in the 8 L bioreactor and also produced three hydrocarbons.

**Conclusions:**

A new oil-rich microalga *S. bacillaris* strain siva2011 was discovered and its biomass has been scaled-up in a newly designed balloon-type bioreactor. The TAGs and hydrocarbons produced by this organism could be used as substrates for jet fuel or biodiesel.

## Background

Worldwide consumption of crude oil is predicted to grow continuously. It is clear that in spite of improvements in the recovery of traditional fossil fuels, alternative renewable energy resources will at some point be needed. Moreover, such renewable fuels offer the prospect of minimizing increases in atmospheric CO_2_ by recycling carbon from the atmosphere back into a viable liquid fuel (or perhaps eventually sequestering it entirely). Over a large number of cycles, the net effect could be a significant reduction in the addition of CO_2_ into the atmosphere compared to continued reliance only on fossil fuels. A wide variety of existing biofuel technologies have been tested, but none have proven to provide a suitable source of replacement liquid fuels. Although current alternatives such as ethanol and biodiesel can provide carbon neutrality, fuels derived largely from normally edible plant sources affect the food supply negatively [1-3]. For these reasons algae feedstocks are being explored as an alternative [4]. The development of a suitable algal-based jet fuel from algal biomass may also impact air transportation.

The jet fuel approach is to chemically process triacylglycerols (TAGs) to alkanes. This could be done by catalytic hydrotreating, breaking the TAG molecule and removing the oxygen to form alkanes. While this product meets diesel specifications, it can be further upgraded into jet fuel or naphtha by hydrocracking, isomerization and catalytic reforming [5]. The by-product propane can be used for residential central heating. However, not all microalgae are capable of producing sufficient TAGs and hydrocarbons for effective fuel production. While others might produce abundant TAGs, they might not necessarily be the optimum TAGs for production of high-value products such as aviation fuel. For production of such specialized fuels, the selection of the algal species is the key to success. Carbon profiles for selecting algal strains [6] and catalytic hydrothermal decarboxylation of fatty acids for aviation fuel [7] have been studied.

Recently, *Stichococcus bacillaris* Naegeli was proposed as a potential and dedicated candidate for use in fuel production [8]. *S. bacillaris* is a green soil microalga which includes over 14 species [9]. Cells are approximately 2 to 3 μm in diameter. The state of filamentous or unicellular structures depends on salinity [10]. This species can grow both in freshwater and seawater with different growth kinetics [11], while tolerating high salinities [12]. In addition, this alga has adapted to low temperatures and is found in ice-free areas of Antarctica [13]. Moreover, it also contains high NADPH-GDH activity [14], low CO_2_ resistance [15] and has unique microtubule organization in prophase [16]. The NADPH-GDH plays an important role in photosynthetic microalgae, which is associated with photoregulation and the incorporation of ammonia into amino acids. The changes in NADPH-GDH were shown in different culture conditions such as photoautotrophic, heterotrophic and mixotrophic [17]. Compared to ammonium, nitrate-grown *S. bacillaris* had higher activity of NADPH-GDH [18]. *S. bacillaris* is fairly abundant globally, can remove heavy metals from hazardous environments [19] and is also capable of biotransforming phenols [20]. These characteristics suggest that *S. bacillaris* could minimize water contamination or improve water quality.

In addition, this organism has a short life cycle and is tolerant to different ranges of pH. Most importantly, over 30% of its dry mass can be produced as oil that can be readily converted to biodiesel [21]. Moreover, this alga produced a high percentage of C16 to C18 carbon fatty acids. Therefore, the goal was to isolate *Stichococcus* species for the study of aviation fuel. Other proposed algal strains either produced triterpene hydrocarbons that are difficult to convert cost-effectively to usable fuels or grew too slowly to be useful [22,23]. Some other TAGs are produced from algae but they typically yield a low biomass [24]. Thus, the aim of this research has been: 1) to isolate new *Stichococcus* algal species producing significant quantities of lipids and hydrocarbons, especially those suitable for production of aviation fuel; and 2) to evaluate the scale-up potential of this alga in a new design balloon-type bioreactor.

## Results and discussions

### *Stichococcus bacillaris* strain siva2011 identification

**Figure 1:**
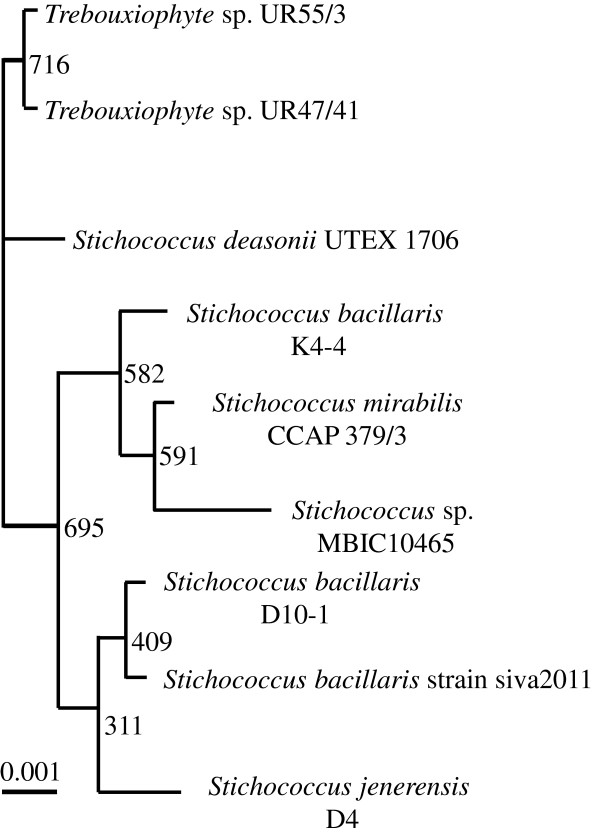
**Phylogenetic tree showing the relationship of *S. bacillaris *strain siva2011 based on 18S rDNA sequences.** A distance of 0.001 is indicated by the scale.

A new axenic microalga *S. bacillaris* strain siva2011 was isolated from an *in vitro* plant. Microscopic examination demonstrated green rod-shaped cells 5 to 10 μm in length and 2 to 3 μm in diameter. The cells are often presented in chains. The 18S sequence data confirmed that this new alga is a strain of genus *Stichococcus* with the greatest similarity to *S. bacillaris*. However, there is a large difference between this and existing strains at nucleotides 610 to 980 of the 18S rDNA (see Additional file 1). The 23S sequence did not match with existing *S. bacillaris* sequences, providing further confirmation that this is a new species. Due to taxonomic problems of the *Stichococcus* species and intraspecific biodiversity [25], this new alga was named ‘*Stichococcus bacillaris* strain siva2011’. The two partial sequences were deposited into the National Center for Biotechnology Information (NCBI) [GenBank:JN168788 and JN168789]. A neighbor-joining tree was created using a Clustal X2.0.12 set to exclude positions with gaps and correct for multiple substitutions. Based on the 18S rDNA sequences, 1,000 bootstrap trials were used to show the relationship of the *S. bacillaris* and the strain siva2011 (Figure 1). Previously, a similar phylogenetic tree was reported for *S. bacillaris* strains NJ-10 and NJ-17 [13].

### Bioreactor culture of *S. bacillaris* strain siva2011

**Figure 2:**
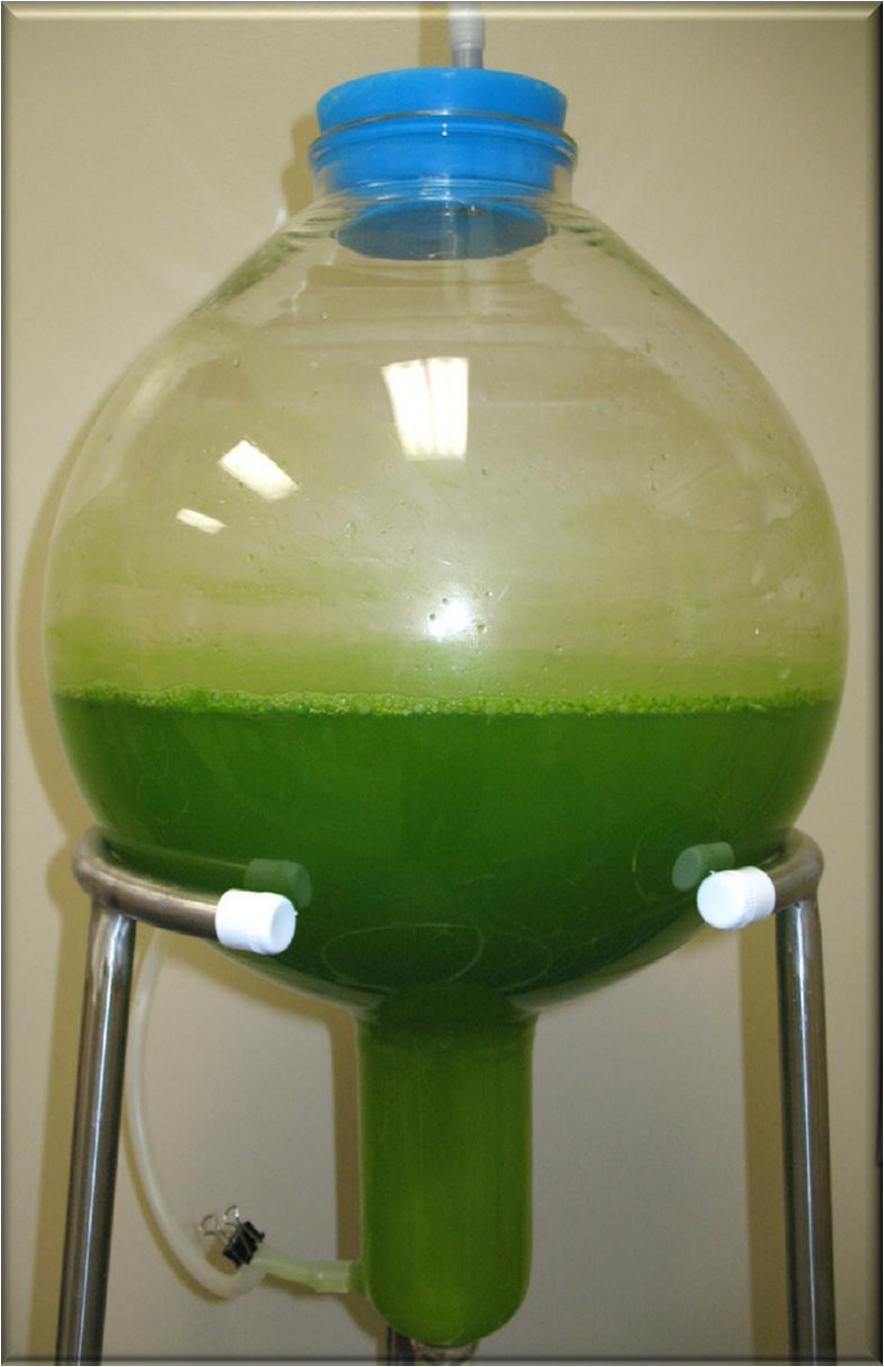
**Balloon-type bioreactor 20 L culture of *S. bacillaris *** **strain siva2011 (working volume 8 L).**

To further explore the potential for enhanced fuel application, production of the *S. bacillaris* strain siva2011 was cultured using a low-cost indoor balloon-type bioreactor (Figure 2). This alga required both light and sugar substrates for optimum growth. The higher biomass accumulation was noticed in 1% fructose supplemented medium when compared to other sugars. It is also important to know that in a 6-day culture, a comparison of the sugar concentrations and the total inorganic carbon in the media showed no significant difference (data not shown). Light limitation is one of the critical factors in algal biomass scale-up in the photobioreactor. This strain grows well under a low light intensity (15 to 30 μE m^-2^ s^-1^ for 10 hours) and requires room temperature. The newly designed reactor has a larger headspace, which efficiently captures light and also has good media circulation to enhance photosynthesis. In other words, this unique bioreactor configuration could minimize the factors limiting the overall rate of photosynthesis in a high density culture.

**Figure 3:**
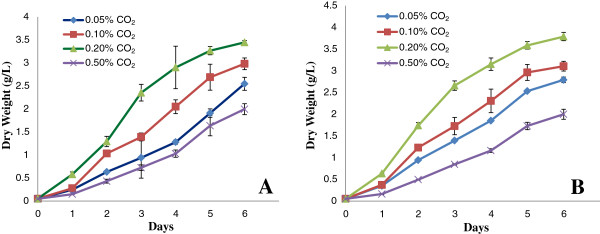
**Growth kinetics of *S. bacillaris *strain siva2011. (A)** Growth for 6 days in a 4 L airlift balloon bioreactor with 0.05, 0.1, 0.2 or 0.5% CO_2_. **(B)** Growth for 6 days in an 8 L airlift balloon bioreactor with 0.05, 0.1, 0.2 or 0.5% CO_2_.

**Figure 4:**
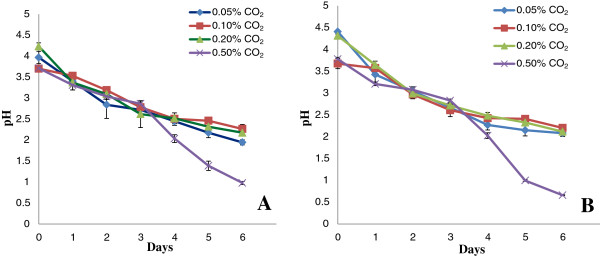
**pH kinetics of *S. bacillaris *strain siva2011. (A)** Grown medium pH for 6 days in a 4 L airlift balloon bioreactor with 0.05, 0.1, 0.2 or 0.5% CO_2_. **(B)** Grown medium pH for 6 days in an 8 L airlift balloon bioreactor with 0.05, 0.1, 0.2 or 0.5% CO_2_.

After autoclaving the media, the pH dropped from 6.0 to 4.2 because of fructose degradation. The sugar degradation could be minimized by sterilizing filtration; however, this procedure increases the percentage of culture contamination. Figure 3A,B illustrates the kinetics of *S. bacillaris* strain siva2011 grown for 6 days in 4 L and 8 L airlift balloon bioreactors with 0.05, 0.1, 0.2 or 0.5% CO_2_, respectively. Of the four concentrations of CO_2_ tested, 0.2% yielded the highest biomass. The exponential growth phase was noticed on day 4 and stationary phase on day 6. Although the culture medium nutrients did not deplete in 6 days, the pH dropped to highly acidic levels (Figure 4A,B). The dropping pH could be caused by the degradation of fructose or CO_2_ effects or HNO_3_ accumulation in the culture medium. For instance, in the aqueous phase, nitrogen oxide from the media could react with oxygen to form nitrogen dioxide, which could then react with the hydroxyl radical to form nitric acid. In order to bring the pH back to an optimum level of 4.5 to 5.0 in fructose supplemented medium, every 6 days the culture was subcultured and old media was removed by centrifugation. Alternatively, adding sodium hydroxide to the culture could perhaps have maintained the pH; further studies are needed for verification. This alga did not grow well in algal Bold’s Basal Medium (BBM) (data not shown).

On day 6 with 0.2% CO_2_, a maximum biomass of 3.79 g/L dry weight (DW) was achieved in 8 L and 3.45 g/L DW in 4 L, respectively. Since the new strain requires a very low 0.2% of CO_2_, the input cost on large-scale could be minimized. When compared to 0.5% CO_2_, algal cells grown in a 4 L bioreactor were higher in biomass, with 0.55, 0.986 and 1.45 g/L DW in the 0.05, 0.1 and 0.2% CO_2_, respectively. Similarly, in the 8 L bioreactor, biomass accumulation was 0.793, 1.107 and 1.79 g/L DW after 6 days of culture. Both growth and pH kinetic trends were similar in 4 L and 8 L bioreactors. The data supports the notion that this strain does not require light intensity over 15 to 30 μE m^-2^ s^-1^; therefore, achieving high density biomass may not be a problem. However, the optimization of air flow is needed for better media circulation and growth. The media circulation is critical for efficient photosynthesis. The oil-rich alga *Ettlia oleoabundans* accumulates 2.28 g/L dry biomass in BBM in approximately 22 to 27 days [24], while *S. bacillaris* strain siva2011 accumulates approximately 1.5 g/L higher biomass within 6 days in modified Murashige and Skoog (MS) medium.

**Figure 5:**
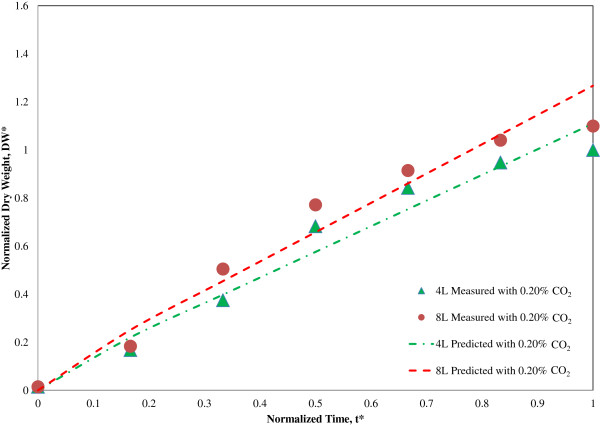
**Prediction of normalized *S. bacillaris *** **strain siva2011 dry weight (DW) for 0.2% CO**_**2 **_**in 4 L and 8 L balloon-type bioreactors.**

For scale-up studies, modeling is necessary because it provides detailed estimates regarding prediction and validation. For instance, DW of *S. bacillaris* strain siva2011 has been regarded as an indicator for productivity in the bioprocess. Prediction of DW is needed and helpful for scale-up of *S. bacillaris* strain siva2011 biomass in large-scale reactors. Quinn *et al*. [26] modeled microalgae growth and lipid accumulation in an outdoor photobioreactor by using MATLAB for biofuel applications. This model qualitatively captures the growth trends with variations in time. Figure 5 shows the prediction of normalized DW for 0.2% CO_2_ in 4 L and 8 L bioreactors compared with experimental/measured data obtained from each test. An overall trend of predicted DW for 8 L was similar to the 4 L data. The R^2^ value has been estimated in the 8 L as 85.8%, while the 4 L exhibits 87.4%. Both tests give an error under 15%. This suggests that the selected variables are not adequate for accurate validation and mapping within an acceptable error. Application of polynomial equations with more variables in the modeling or another mathematical approach might be warranted for future optimization if better accuracy is needed.

### Hydrocarbons and FAMEs

**Figure 6:**
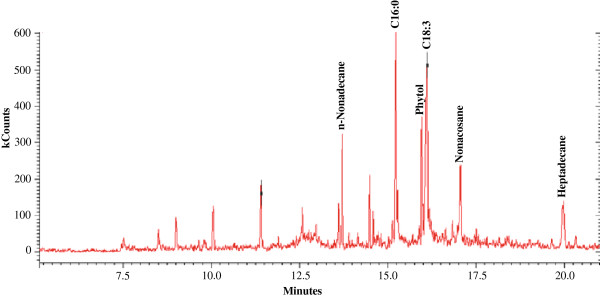
**GC-MS profile of the hydrocarbons and free fatty acids from *S. bacillaris *strain siva2011 biomass.** GC-MS, gas chromatography–mass spectrometry.

Figure 6 shows the gas chromatography–mass spectrometry (GC-MS) total ion chromatogram of the hydrocarbon fraction harvested from *S. bacillaris* strain siva2011 biomass. This chromatogram shows that this strain biosynthesizes three free hydrocarbons, namely n-nonadecane (C_19_H_40_), nonacosane (C_29_H_60_) and heptadecane (C_17_H_36_). These alkanes are also found in traditional and non-traditional liquid fuels. The *S. bacillaris* strain siva2011 contained 1.36% of total hydrocarbons: the C_19_H_40_, C_29_H_60_ and C_17_H_36_ were 6.3, 4.1 and 3.2 mg/g DW, respectively, with 0.2% CO_2_. In addition, it was unchanged in both the 4 L and 8 L bioreactor studies. Some of these hydrocarbons were previously reported in other algal species [27,28]. In cyanobacteria, a two-step alkane biosynthetic pathway was reported: 1) acyl-acyl carrier protein (ACP) reductase converted ACP into aldehyde by reduction; and 2) an aldehyde-deformylating oxygenase converted aldehyde into alkane or alkene by oxidation [29,30]. The overexpressed ACP reductase and aldehyde-deformylating oxygenase cyanobacteria LX56 strain biomass accumulated 1.1% DW of alkane in a column photobioreactor [30]. In general, alkane accumulation was toxic to algal cells, therefore, production was lowered. However, the longer chain (over C12) of alkane accumulation was insignificant with respect to toxicity in *Saccharomyces cerevisiae* cells, while C8 to C11 alkanes were cytotoxic [31]. Although the *S. bacillaris* strain siva2011 biosynthesize longer chain hydrocarbons, the alkane level is lower than in the TAGs. Since the *S. bacillaris* strain siva2011 is a new species, a reference genome is necessary for transcriptome analysis, and subsequent metabolic engineering is unavailable.

**Figure 7:**
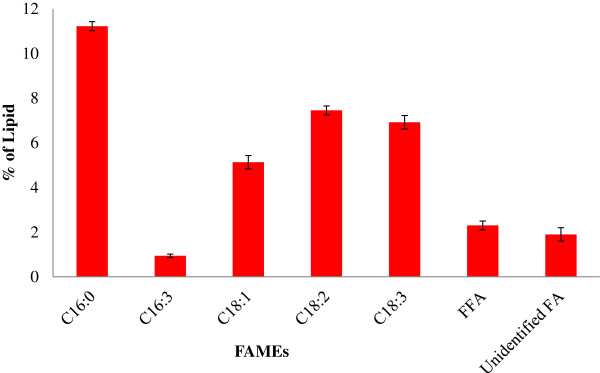
**Total lipid content from *S. bacillaris *strain siva2011, biomass from 20 L bioreactor and working volume 8 L with 0.2% CO**_**2 **_**on day 6.** FA, fatty acid; FAME, fatty acid methyl ester; FFA, free fatty acid.

The *S. bacillaris* strain siva2011 biomass contains two free fatty acids (FFAs) (palmitic acid (C16:0), linolenic acid (C18:3)) and phytol (Figure 6). Figure 7 illustrates the fatty acid methyl ester (FAME) profile and total lipid (35.82%) content of *S. bacillaris* strain siva2011 biomass from the 8 L bioreactor culture with 0.2% CO_2_. This data shows that this strain contains 31.62% of total FAMEs, 2.3% of FFAs and 1.9% of unidentified fatty acids, which is higher compared to the 4 L bioreactor. In addition, 1.2% of phytol was also found. The *S. bacillaris* strain 158/11 biomass contained 32% of total lipid and 1% of phytol [21]. The results show that this strain contains a high degree of unsaturated fatty acids. The main unsaturated FAMEs detected are methyl hexadecatrienoate (C16:3), methyl oleate (C18:1), methyl linoleate (C18:2) and methyl linolenate (C18:3). The predominant saturated FAME is methyl palmitate (C16:0). This profile is consistent with the other *S. bacillaris* strains, NJ-10 and NJ-17 [13]. When *S. bacillaris* strain siva2011 was scaled-up in the 8 L bioreactor with 0.2% CO_2_, the C16:0, C16:3, C18:1, C18:2 and C18:3 were 112.2, 9.4, 51.3, 74.1 and 69.2 mg/g DW, respectively; in the 4 L bioreactor the FAMEs were 102, 8.1, 49.4, 71.7 and 65.3 mg/g, respectively, which is higher compared to the 0.5% CO_2_. It suggests not only that *S. bacillaris* strain siva2011 biomass was scaled-up, but also that the TAG production metabolism appeared to be scaled-up as well. However, the FAME profiles were unchanged. Previously this reactor was used for plant root culture and was successful at commercial-scale (10,000 L) cultivation of small molecules [32,33]. It was also demonstrated that the resveratrol metabolic pathway gene, chalcone synthase, was highly expressed in this type of reactor. Moreover, this reactor design has not only non-agitation hydrodynamics but also enhanced geometry, flow dynamics and kinematics [33,34]. Thus, this configuration could enhance light capturing by mixing the algal cells evenly, which facilitates photosynthesis and biomass accumulation as well as possibly up-regulating fatty acid pathway genes. Olivieri *et al*. [21] showed that the *S. bacillaris* 158/11 contains C16:0 6.5%, C18:3 5.2%, C18:2 4.6% and C18:1 13.8%. This suggests that the *Stichococcus* species has unique fatty acid profiles which could be used for high-quality liquid fuel production. However, the actual large-scale feasibility test for algal biomass scale-up is needed for aviation fuel production.

## Conclusions

Long-term energy demands will eventually greatly outweigh the world supply of fossil fuels, and their use increases greenhouse gases. Therefore, alternative sources and methods of producing fuels must be found. Although algae can capture greenhouse gas emissions while producing oxygen, the need for high biomass and oil accumulation are challenging for algal-based bioenergy production. *S. bacillaris* strain siva2011 is rich in lipids, presumably TAGs, with a suitable carbon range for aviation or other liquid fuels. Indeed, scaling studies showed that at 0.2% CO_2_ supplementation *S. bacillaris* strain siva2011 had better growth and increased FAMEs in the 8 L bioreactor than 4 L. It is likely that the 20 L bioreactor could have substantially lower hydrodynamic stress. However, further studies on mass transfer at larger scales seem to be warranted. The culture conditions vary from alga species to species. *S. bacillaris* strain siva2011 can grow in conditions mentioned in this study. Irrespective of the need to further characterize the biochemical pathways for this organism, it is nevertheless important to point out that there is already sufficient empirical evidence that it will likely be a possible candidate for renewable production of light liquid fuels based on the copious production of lipids and hydrocarbons, and especially the relatively high degree of unsaturation found therein.

## Materials and methods

### Isolation and identification of *Stichococcus bacillaris* strain siva2011

Axenic *S. bacillaris* strain siva2011 cells were isolated from *in vitro Lagerstroemia* seedlings. The morphology of algal cells was identified by light microscopy. The genus and species were identified by the 18S rDNA region of the nuclear chromosome and the 23S region of the chloroplast rDNA. PCR was performed using primers to amplify the 23S and the 18S region of the rDNA. The products were then sequenced. The genus *Stichococcus* was identified based on the 18S rDNA sequence in the NCBI database. The identification was confirmed and authenticated by The Culture Collection of Algae at the University of Texas at Austin (UTEX), Austin, TX, USA. The phylogenetic tree was created based on the 18S rDNA sequence using a Clustal X2.0.12 set to exclude positions with gaps, correct for multiple substitutions and run 1,000 bootstrap trials.

### Bioreactor culture

The new alga *S. bacillaris* strain siva2011 was cultured in 5 L and 20 L liquid-phase airlift balloon-type bioreactors [32-34] with modified MS [35] liquid medium for 6 days. This alga was also tested in BBM for comparison [36]. To evaluate the scale-up potential of the balloon-type bioreactor for larger-scale use, the 5 L bioreactor was used for the 4 L working volume and a 20 L bioreactor was used for the 8 L working volume in order to gain a linear biomass pattern for prediction or modeling. Working volumes of bioreactors for scale-up studies were previously published [37] for root culture and were not repeated here. Balloon bioreactors have a larger headspace. The 5 L bioreactor has an 8 inch diameter and the 20 L has a 12 inch diameter, which may facilitate efficient light absorption and medium circulation for algal culture. The modified MS medium contains reduced NH_4_NO_3_ 0.6 g, KNO_3_ 1.5 g [32,33] with 1% fructose and pH 6.0. The cool white fluorescent room lights at 15 to 30 μE m^-2^ s^-1^ for 10 hours followed by 14 hours of dark and 23 to 25°C culture conditions were used. After autoclaving the medium and the bioreactor, the axenic algal cells were cultured into the bioreactor. The inoculum was active cells that were 3 days old and 0.05 g fresh weight (FW)/L. The bioreactor cultures were supplemented with different concentrations of sterile filtered CO_2_ such as 0.05, 0.1, 0.2 or 0.5%. The input air mixture CO_2_ gas flow was set at 0.1 vvm (volume (of air) per volume (of liquid) per minute). To screen growth kinetics of *S. bacillaris* strain siva2011, algal biomass was harvested, and medium pH was measured after 1, 2, 3, 4, 5 and 6 days. Algal cells were harvested by centrifugation at 10,000 rpm for 5 minutes. After harvesting, algal biomass were frozen in liquid nitrogen and freeze-dried. DW was recorded after the samples were freeze-dried to a constant weight.

### Linear regression

A linear regression model has been developed based on 0.2% CO_2_ experimental data to predict DW of *S. bacillaris* strain siva2011 production ranging from 4 L to 8 L. Related variables including DW, yCO_2_, pH, time (t) and volume (V) are listed from constituents in *S. bacillaris* strain siva2011 production tests, as follows:

(1) $$\mathrm{DW}=f\left(\mathrm{yC}O_{2},\mathrm{pH},t,V\right)$$

To test its feasibility, it has been simplified into a linear equation with two variables, t and V, among the whole variables as shown in Equation (2):

(2) $$\begin{array}{c} \mathrm{DW}=f\left(t,V\right)\\ \;=\left(\mathrm{at}+b\right)\cdot V^{c} \end{array}$$

Experimental data and variables have been normalized using maximum DW and t to establish a mathematical model as shown in Equations (3) and (4).

(3) $$DW^{*}=\mathrm{DW}/DW_{f}$$

(4) $$t^{*}=t/t_{f}$$

Where DW_f_ is maximum DW (g/L) produced from 8 L test under 0.2% CO_2_ fraction and t_f_ is maximum t to reach the maximum DW. The DW_f_ and t_f_ were 3.79 g/L on day 6. V has been standardized by a baseline 8 L in this model. Constants a, b and c in Equation (2) have been determined using test data and a linear least squares method. The 4 L and 8 L data were used to determine the constants: a, b and c. The final non-dimensional equation is suggested by Equation (5):

(5) $$DW^{*}=\left(1.0676\cdot t^{*}+0.042\right)\cdot {\left(\frac{V_{2}}{V_{1}}\right)}^{0.19177}$$

V_1_ was base volume at 4 L and V_2_ was extended at 8 L volume.

### Analysis of hydrocarbons and FAMEs

One gram of 6-day-old freeze-dried algal cells were used for analysis of hydrocarbons and FAMEs. The total lipids were evaluated according to Jones *et al*. [38]. FAMEs were processed according to the AOAC method 996.06 and AOCS method Ce 1 h-05 [39,40]. Each FAME GC-MS spectra were acquired using a Clarus 500 gas chromatography (PerkinElmer, Waltham, MA, USA) coupled to a Clarus A mass spectrometer (PerkinElmer). A FAMEWAX column (Restek, Bellefonte, PA, USA) was used for separation of FAMEs (30 m length, 0.25 mm ID, 0.25 μm film thickness). The column conditions were determined prior to analysis using a FAME and hydrocarbon reference mixture. Initially, the gas chromatography temperature was 30°C and ramped 10°C/min to a final temp of 220°C and held for 15 minutes at 220°C. Helium was used as the carrier gas. The flow rate was set at 1 mL/min and the spilt ratio was 1:20. The sample injection volume was 1 μL. The mass spectrometer was set to record ranges of spectra from 50 to 500 m/z. The inlet line temperature was set at 300°C and the source temperature was 180°C. Hydrocarbons were processed and analyzed in GC-MS according to Wang *et al*. [30]. Quantitative analysis of hydrocarbons and FAMEs in the algal biomass was calculated from the calibration curve of the respective standard. Data acquisition and processing were performed with TurboMass software (PerkinElmer).

### Statistical analysis

All experiments were repeated at least three times, each with three replications except sequencing. The experimental variations were expressed as a mean standard error.

## Abbreviations

ACP: Acyl-acyl carrier protein; BBM: Bold’s Basal Medium; CO2: Carbon dioxide; DW: Dry weight; FA: Fatty acid; FAME: Fatty acid methyl ester; FFA: Free fatty acid; FW: Fresh weight; GC-MS: Gas chromatography–mass spectrometry; GDH: Glutamate dehydrogenase; HNO3: Nitric acid; KNO3: Potassium nitrate; MS: Murashige and Skoog; NADPH: Nicotinamide adenine dinucleotide phosphate; NCBI: National Center for Biotechnology Information; NH4NO3: Ammonium nitrate; PCR: Polymerase chain reaction; TAG: Triacylglycerol; UTEX: The Culture Collection of Algae at the University of Texas at Austin.

## Competing interests

The authors declare that they have no competing interests.

## Authors’ contributions

GS, JL and KJ made substantial contributions to experimental design or analysis or interpretation of data. Specifically, GS led and designed the experiments and performed the organism isolation, bioprocessing and GC-MS quantitative studies. JL performed the gas chromatography separation and mass spectrometry experiments for compound identification. KJ performed the mathematical model to validate the variables. GS wrote the manuscript, which was reviewed and approved by all authors. All authors read and approved the final manuscript.

## Supplementary Material

Additional file 1 **NCBI basic local alignment search tool comparison of the *S. bacillaris *strain siva2011 18S rDNA sequences with different *Stichococcus* species.** A large difference was found in the Clustal X2.0.12 multiple sequence alignment between the *S. bacillaris* strain siva2011 and the existing strains at nucleotides 610 to 980. SIVA, *S. bacillaris* strain siva2011. Click here for file
