# Supplementary material for: Bioprocessing of Stichococcus bacillaris strain siva2011
Source: Biotechnol Biofuels. 2014 Apr 15;7:62. doi: 10.1186/1754-6834-7-62 (PMC4022374; doi:10.1186/1754-6834-7-62)

Additional file 1

```
              ***************** ***  *******************************************************************************************************************************
S_bacillaris  TGCATGTCTAAGTATAAACTGCTTTATACTGTGAAACTGCGAATGGCTCATTAAATCAGTTATAGTTTATTTGATGGTACCTTACTACTCGGATAACCGTAGTAATTCTAGAGCTAATACGTGCGCAAATCCCGACTTCCGGAAGGGACG  150
S_jenerensis  TGCATGTCTAAGTATAAACTGCTTTATACTGTGAAACTGCGAATGGCTCATTAAATCAGTTATAGTTTATTTGATGGTACCTTACTACTCGGATAACCGTAGTAATTCTAGAGCTAATACGTGCGCAAATCCCGACTTCCGGAAGGGACG  150
S_mirabilis   TGCATGTCTAAGTATAAACTGCTTTATACTGTGAAACTGCGAATGGCTCATTAAATCAGTTATAGTTTATTTGATGGTACCTTACTACTCGGATAACCGTAGTAATTCTAGAGCTAATACGTGCGCAAATCCCGACTTCCGGAAGGGACG  150
S_sp          TGCATGTCTAAGTATAAGCTGACTTATACTGTGAAACTGCGAATGGCTCATTAAATCAGTTATAGTTTATTTGATGGTACCTTACTACTCGGATAACCGTAGTAATTCTAGAGCTAATACGTGCGCAAATCCCGACTTCCGGAAGGGACG  150
S_bacillaris2 TGCATGTCTAAGTATAAACTGCTTTATACTGTGAAACTGCGAATGGCTCATTAAATCAGTTATAGTTTATTTGATGGTACCTTACTACTCGGATAACCGTAGTAATTCTAGAGCTAATACGTGCGCAAATCCCGACTTCCGGAAGGGACG  150
Treb_sp1      TGCATGTCTAAGTATAAACTGCTTTATACTGTGAAACTGCGAATGGCTCATTAAATCAGTTATAGTTTATTTGATGGTACCTTACTACTCGGATAACCGTAGTAATTCTAGAGCTAATACGTGCGCAAATCCCGACTTCCGGAAGGGACG  150
Treb_sp2      TGCATGTCTAAGTATAAACTGCTTTATACTGTGAAACTGCGAATGGCTCATTAAATCAGTTATAGTTTATTTGATGGTACCTTACTACTCGGATAACCGTAGTAATTCTAGAGCTAATACGTGCGCAAATCCCGACTTCCGGAAGGGACG  150
S_deasonii    TGCATGTCTAAGTATAAACTGCTTTATACTGTGAAACTGCGAATGGCTCATTAAATCAGTTATAGTTTATTTGATGGTACCTTACTACTCGGATAACCGTAGTAATTCTAGAGCTAATACGTGCGCAAATCCCGACTTCCGGAAGGGACG  150
SIVA          TGCATGTCTAAGTATAAACTGCTTTATACTGTGAAACTGCGAATGGCTCATTAAATCAGTTATAGTTTATTTGATGGTACCTTACTACTCGGATAACCGTAGTAATTCTAGAGCTAATACGTGCGCAAATCCCGACTTCCGGAAGGGACG  150
              1.......10........20........30........40........50........60........70........80........90.......100.......110.......120.......130.......140.......150

              ****************************************************************************  ************************************************************************
S_bacillaris  TATTTATTAGATAAAAGGCCGACCGGGCTTGCCCGACTCGCGGTGAATCATGATAACTTCACGAATCGCATGGCCTCCGCGCCGGCGATGTTTCATTCAAATTTCTGCCCTATCAACTTTCGATGGTAGGATAGAGGCCTACCATGGTGG  300
S_jenerensis  TATTTATTAGATAAAAGGCCGACCGGGCTTGCCCGACTCGCGGTGAATCATGATAACTTCACGAATCGCATGGCCTCCGCGCCGGCGATGTTTCATTCAAATTTCTGCCCTATCAACTTTCGATGGTAGGATAGAGGCCTACCATGGTGG  300
S_mirabilis   TATTTATTAGATAAAAGGCCGACCGGGCTTGCCCGACTCGCGGTGAATCATGATAACTTCACGAATCGCATGGCCTTCGCGCCGGCGATGTTTCATTCAAATTTCTGCCCTATCAACTTTCGATGGTAGGATAGAGGCCTACCATGGTGG  300
S_sp          TATTTATTAGATAAAAGGCCGACCGGGCTTGCCCGACTCGCGGTGAATCATGATAACTTCACGAATCGCATGGCCTCCGCGCCGGCGATGTTTCATTCAAATTTCTGCCCTATCAACTTTCGATGGTAGGATAGAGGCCTACCATGGTGG  300
S_bacillaris2 TATTTATTAGATAAAAGGCCGACCGGGCTTGCCCGACTCGCGGTGAATCATGATAACTTCACGAATCGCATGGCCTA-GCGCCGGCGATGTTTCATTCAAATTTCTGCCCTATCAACTTTCGATGGTAGGATAGAGGCCTACCATGGTGG  299
Treb_sp1      TATTTATTAGATAAAAGGCCGACCGGGCTTGCCCGACTCGCGGTGAATCATGATAACTTCACGAATCGCATGGCCTCCGCGCCGGCGATGTTTCATTCAAATTTCTGCCCTATCAACTTTCGATGGTAGGATAGAGGCCTACCATGGTGG  300
Treb_sp2      TATTTATTAGATAAAAGGCCGACCGGGCTTGCCCGACTCGCGGTGAATCATGATAACTTCACGAATCGCATGGCCTCCGCGCCGGCGATGTTTCATTCAAATTTCTGCCCTATCAACTTTCGATGGTAGGATAGAGGCCTACCATGGTGG  300
S_deasonii    TATTTATTAGATAAAAGGCCGACCGGGCTTGCCCGACTCGCGGTGAATCATGATAACTTCACGAATCGCATGGCCTTTGCGCCGGCGATGTTTCATTCAAATTTCTGCCCTATCAACTTTCGATGGTAGGATAGAGGCCTACCATGGTGG  300
SIVA          TATTTATTAGATAAAAGGCCGACCGGGCTTGCCCGACTCGCGGTGAATCATGATAACTTCACGAATCGCATGGCCTTTGCGCCGGCGATGTTTCATTCAAATTTCTGCCCTATCAACTTTCGATGGTAGGATAGAGGCCTACCATGGTGG  300
              .......160.......170.......180.......190.......200.......210.......220.......230.......240.......250.......260.......270.......280.......290.......300

              ******************************************************************************************************************************************************
S_bacillaris  TAACGGGTGACGGGGGATTAGGGTTCGATTCCGGAGAGGGAGCCTGAGAAACGGCTACCACATCCAAGGAAGGCAGCAGGCGCGCAAATTACCCAATCCTGACACAGGGAGGTAGTGACAATAAATAACAATACCGGGCTTTTCAAGTCT  450
S_jenerensis  TAACGGGTGACGGGGGATTAGGGTTCGATTCCGGAGAGGGAGCCTGAGAAACGGCTACCACATCCAAGGAAGGCAGCAGGCGCGCAAATTACCCAATCCTGACACAGGGAGGTAGTGACAATAAATAACAATACCGGGCTTTTCAAGTCT  450
S_mirabilis   TAACGGGTGACGGGGGATTAGGGTTCGATTCCGGAGAGGGAGCCTGAGAAACGGCTACCACATCCAAGGAAGGCAGCAGGCGCGCAAATTACCCAATCCTGACACAGGGAGGTAGTGACAATAAATAACAATACCGGGCTTTTCAAGTCT  450
S_sp          TAACGGGTGACGGGGGATTAGGGTTCGATTCCGGAGAGGGAGCCTGAGAAACGGCTACCACATCCAAGGAAGGCAGCAGGCGCGCAAATTACCCAATCCTGACACAGGGAGGTAGTGACAATAAATAACAATACCGGGCTTTTCAAGTCT  450
S_bacillaris2 TAACGGGTGACGGGGGATTAGGGTTCGATTCCGGAGAGGGAGCCTGAGAAACGGCTACCACATCCAAGGAAGGCAGCAGGCGCGCAAATTACCCAATCCTGACACAGGGAGGTAGTGACAATAAATAACAATACCGGGCTTTTCAAGTCT  449
Treb_sp1      TAACGGGTGACGGGGGATTAGGGTTCGATTCCGGAGAGGGAGCCTGAGAAACGGCTACCACATCCAAGGAAGGCAGCAGGCGCGCAAATTACCCAATCCTGACACAGGGAGGTAGTGACAATAAATAACAATACCGGGCTTTTCAAGTCT  450
Treb_sp2      TAACGGGTGACGGGGGATTAGGGTTCGATTCCGGAGAGGGAGCCTGAGAAACGGCTACCACATCCAAGGAAGGCAGCAGGCGCGCAAATTACCCAATCCTGACACAGGGAGGTAGTGACAATAAATAACAATACCGGGCTTTTCAAGTCT  450
S_deasonii    TAACGGGTGACGGGGGATTAGGGTTCGATTCCGGAGAGGGAGCCTGAGAAACGGCTACCACATCCAAGGAAGGCAGCAGGCGCGCAAATTACCCAATCCTGACACAGGGAGGTAGTGACAATAAATAACAATACCGGGCTTTTCAAGTCT  450
SIVA          TAACGGGTGACGGGGGATTAGGGTTCGATTCCGGAGAGGGAGCCTGAGAAACGGCTACCACATCCAAGGAAGGCAGCAGGCGCGCAAATTACCCAATCCTGACACAGGGAGGTAGTGACAATAAATAACAATACCGGGCTTTTCAAGTCT  450
              .......310.......320.......330.......340.......350.......360.......370.......380.......390.......400.......410.......420.......430.......440.......450

              ************************** ********************************************** ****************************************************************************
S_bacillaris  GGTAATTGGAATGAGTACAATCTAAATCCCTTAACGAGGATCAATTGGAGGGCAAGTCTGGTGCCAGCAGCCGCGGTAATTCCAGCTCCAATAGCGTATATTTAAGTTGCTGCAGTTAAAAAGCTCGTAGTTGGATTTCGGATGGGTTCC  600
S_jenerensis  GGTAATTGGAATGAGTACAATCTAAATCCCTTAACGAGGATCAATTGGAGGGCAAGTCTGGTGCCAGCAGCCGCGGTAATTCCAGCTCCAATAGCGTATATTTAAGTTGCTGCAGTTAAAAAGCTCGTAGTTGGATTTCGGATGGGTTCC  600
S_mirabilis   GGTAATTGGAATGAGTACAATCTAAATCCCTTAACGAGGATCAATTGGAGGGCAAGTCTGGTGCCAGCAGCCGCGGTAATTCCAGCTCCAATAGCGTATATTTAAGTTGCTGCAGTTAAAAAGCTCGTAGTTGGATTTCGGATGGGTTCC  600
S_sp          GGTAATTGGAATGAGTACAATCTAAATCCCTTAACGAGGATCAATTGGAGGGCAAGTCTGGTGCCAGCAGCCGCGGTAATTCCAGCTCCAATAGCGTATATTTAAGTTGCTGCAGTTAAAAAGCTCGTAGTTGGATTTCGGATGGGTTCC  600
S_bacillaris2 GGTAATTGGAATGAGTACAATCTAAACCCCTTAACGAGGATCAATTGGAGGGCAAGTCTGGTGCCAGCAGCCGCGGTAATTCCAGCTCCAATAGCGTATATTTAAGTTGCTGCAGTTAAAAAGCTCGTAGTTGGATTTCGGATGGGTTCC  599
Treb_sp1      GGTAATTGGAATGAGTACAATCTAAATCCCTTAACGAGGATCAATTGGAGGGCAAGTCTGGTGCCAGCAGCCGCGGTAATTCCAGCTCCAATAGCGTATATTTAAGTTGCTGCAGTTAAAAAGCTCGTAGTTGGATTTCGGATGGGTTCC  600
Treb_sp2      GGTAATTGGAATGAGTACAATCTAAATCCCTTAACGAGGATCAATTGGAGGGCAAGTCTGGTGCCAGCAGCCGNGGTAATTCCAGCTCCAATAGCGTATATTTAAGTTGCTGCAGTTAAAAAGCTCGTAGTTGGATTTCGGATGGGTTCC  600
S_deasonii    GGTAATTGGAATGAGTACAATCTAAATCCCTTAACGAGGATCAATTGGAGGGCAAGTCTGGTGCCAGCAGCCGCGGTAATTCCAGCTCCAATAGCGTATATTTAAGTTGCTGCAGTTAAAAAGCTCGTAGTTGGATTTCGGATGGGTTCC  600
SIVA          GGTAATTGGAATGAGTACAATCTAAATCCCTTAACGAGGATCAATTGGAGGGCAAGTCTGGTGCCAGCAGCCGCGGTAATTCCAGCTCCAATAGCGTATATTTAAGTTGCTGCAGTTAAAAAGCTCGTAGTTGGATTTCGGATGGGTTCC  600
              .......460.......470.......480.......490.......500.......510.......520.......530.......540.......550.......560.......570.......580.......590.......600
```

Additional file 1 continuation

```
             * ********** *  ********* * ***************** ********************* * ********** ******** ************************************* ***********************
S_bacillaris  GCCGGTCCGCCGTTTCGGTGTGCACTGACGGCGCCCATCTTGCTGCCGGGGACGGGCTCCTGGGCTTCACTGTCCGGGACTCGGAGTCGGCGAGGTTACTTTGAGTAAATTAGAGTGTTCAAAGCAGGCCTACGCTCTGAATACATTAGC   750
S_jenerensis  GCCGGTCCGCCGTTTCGGTGTGCACTGACGGCGCCCATCTTGCTGCCGGGGACGGGCTCCTGGGCTTAATTGTCCGGGACTCGGAGTCGGCGAGGTTACTTTGAGTAAATTAGAGTGTTCAAAGCAGGCCTACGCTCTGAATACATTAGC   750
S_mirabilis   GTCGGTCCGCCGTTTCGGTGTGCACTGGCGGCGCCCATCTTGCTGCCGGGGACGGGCTCCTGGGCTTCACTGTCCGGGACCCGGAGTCGGCGAGGTTACTTTGAGTAAATTAGAGTGTTCAAAGCAGGCCTACGCTCTGAATACATTAGC   750
S_sp          GTCGGTCCGCCGCTTCGGTGTGCACCGGCGGCGCCCATCTTGCTGCCGGGGACGGGCTCCTGGGCTTCACTGTCCGGGACTCGGAGTCGGCGAGGTTACTTTGAGTAAATTAGAGTGTTCAAAGCAGGCCTACGCTCTGAATACATTAGC   750
S_bacillaris2 GTCGGTCCGCCGTTTCGGTGTGCACTGGCGGCGCCCATCTTGCTGTCGGGGACGGGCTCCTGGGCTTCACTGTCCGGGACTCGGAGTCGACGAGGTTACTTTGAGTAAATTAGAGTGTTCAAAGCAGGCCTACGCTCTGAATACATTAGC   749
Treb_sp1      GCCGGTCCGCCGTTTCGGTGTGCACTGACGGCGCCCATCTTGCTGTCGGGGACGGGCTCCTGGGCTTAACTGTCCGGGACTCGGAGTCGACGAGGTTACTTTGAGTAAATTAGAGTGTTCAAAGCAGGCCTACGCTCTGAATACATTAGC   750
Treb_sp2      GCCGGTCCGCCGTTTCGGTGTGCACTGACGGCGCCCATCTTGCTGTCGGGGACGGGCTCCTGGGCTTAACTGTCCGGGACTCGGAGTCGACGAGGTTACTTTGAGTAAATTAGAGTGTTCAAAGCAGGCCTACGCTCTGAATACATTAGC   750
S_deasonii    GCCGGTCCGCCGTTTCGGTGTGCACTGACGGCGCCCATCTTGCTGTCGGGGACGGGCTCCTGGGCTTAACTGTCCGGGACTCGGAGTCGACGAGGTTACTTTGAGTAAATTAGAGTGTTCAAAGCAGGCCTACGCTCTGAATACATTAGC   750
SIVA          GCCGGTCCGCCGTTCAGGTGTGCACTGACGGCGCCCATCTTGCTGCCGGGGACGGGCTCCTGGGCTTCACTGTCCGGGACTCGGAGTCGGCGAGGTTACTTTGAGTAAATTAGAGTGTTCAAAGCANGCCTACGCTCTGAATACATTAGC   750
              .......610.......620.......630.......640.......650.......660.......670.......680.......690.......700.......710.......720.......730.......740.......750
```

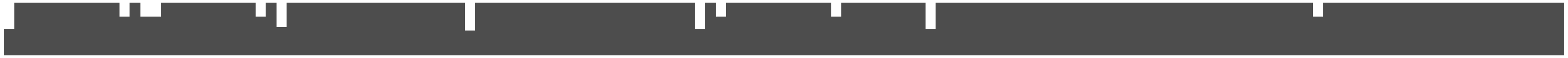

```
              ******************************************** ****************** ********** ************************* ************** **********************************
S_bacillaris  ATGGAATAACACGATAGGACTCTGGCCTATCTTGTTGGTCTGTAGGACCGGAGTAATGATTAAGAGGGACAGTCGGGGGCATTCGTATTTCATTGTCAGAGGTGAAATTCTTGGATTTATGAAAGACGAACTACTGCGAAAGCATTTGCC   900
S_jenerensis  ATGGAATAACACGATAGGACTCTGGCCTATCTTGTTGGTCTGTAGGACCGGAGTAATGATTAAGAGGGACAGTCGGGGGCATTCGTATTTCATTGTCAGAGGTGAAATTCTTGGATTTATGAAAGACGAACTACTGCGAAAGCATTTGCC   900
S_mirabilis   ATGGAATAACACGATAGGACTCTGGCCTATCTTGTTGGTCTGTAGGACCGGAGTAATGATTAAGAGGGACAGTCGGGGGCATTCGTATTTCATTGTCAGAGGTGAAATTCTTGGATTTATGAAAGACGAACTACTGCGAAAGCATTTGCC   900
S_sp          ATGGAATAACACGATAGGACTCTGGCCTATCTTGTTGGTCTGTAGGACCGGAGTAATGATTAAGAGGGACAGTCGGGGGCATTCGTATTTCATTGTCAGAGGTGAAATTCTTGGATTTATGAAAGACGAACTACTGCGAAAGCATTTGCC   900
S_bacillaris2 ATGGAATAACACGATAGGACTCTGGCCTATCTTGTTGGTCTGTAGGACCGGAGTAATGATTAAGAGGGACAGTCGGGGGCATTCGTATTTCATTGTCAGAGGTGAAATTCTTGGATTTATGAAAGACGAACTACTGCGAAAGCATTTGCC   899
Treb_sp1      ATGGAATAACACGATAGGACTCTGGCCTATCTTGTTGGTCTGTAGGACCGGAGTAATGATTAAGAGGGACAGTCGGGGGCATTCGTATTTCATTGTCAGAGGTGAAATTCTTGGATTTATGAAAGACGAACTACTGCGAAAGCATTTGCC   900
Treb_sp2      ATGGAATAACACGATAGGACTCTGGCCTATCTTGTTGGTCTGTAGGACCGGAGTAATGATTAAGAGGGACAGTCGGGGGCATTCGTATTTCATTGTCAGAGGTGAAATTCTTGGATTTATGAAAGACGAACTACTGCGAAAGCATTTGCC   900
S_deasonii    ATGGAATAACACGATAGGACTCTGGCCTATCTTGTTGGTCTGTAGGACCGGAGTAATGATTAAGAGGGACAGTCGGGGGCATTCGTATTTCATTGTCAGAGGTGAAATTCTTGGATTTATGAAAGACGAACTACTGCGAAAGCATTTGCC   900
SIVA          ATGGAATAACACGATAGGACTCTGGCCTATCTTGTTGGTCTGTANGACCGGAGTAATGATTAANAGGGACAGTCNGGGGCATTCGTATTTCATTGTCAGANGTGAAATTCTTGGANTTATGAAAGACGAACTACTGCGAAAGCATTTGCC   900
              .......760.......770.......780.......790.......800.......810.......820.......830.......840.......850.......860.......870.......880.......890.......900
```

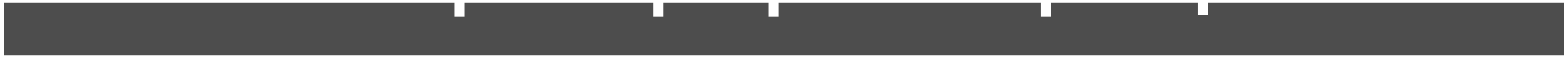

```
              ************ ************************************** *********** ******** **********
S_bacillaris  AAGGATGTTTTCATTAATCAAGAACGAAAGTTGGGGGCTCGAAGACGATTAGATACCGTCCTAGTCTCAACCATAAACGATGC   983
S_jenerensis  AAGGATGTTTTCATTAATCAAGAACGAAAGTTGGGGGCTCGAAGACGATTAGATACCGTCCTAGTCTCAACCATAAACGATGC   983
S_mirabilis   AAGGATGTTTTCATTAATCAAGAACGAAAGTTGGGGGCTCGAAGACGATTAGATACCGTCCTAGTCTCAACCATAAACGATGC   983
S_sp          AAGGATGTTTTCATTAATCAAGAACGAAAGTTGGGGGCTCGAAGACGATTAGATACCGTCCTAGTCTCAACCATAAACGATGC   983
S_bacillaris2 AAGGATGTTTTCATTAATCAAGAACGAAAGTTGGGGGCTCGAAGACGATTAGATACCGTCCTAGTCTCAACCATAAACGATGC   982
Treb_sp1      AAGGATGTTTTCATTAATCAAGAACGAAAGTTGGGGGCTCGAAGACGATTAGATACCGTCCTAGTCTCAACCATAAACGATGC   983
Treb_sp2      AAGGATGTTTTCATTAATCAAGAACGAAAGTTGGGGGCTCGAAGACGATTAGATACCGTCCTAGTCTCAACCATAAACGATGC   983
S_deasonii    AAGGATGTTTTCATTAATCAAGAACGAAAGTTGGGGGCTCGAAGACGATTAGATACCGTCCTAGTCTCAACCATAAACGATGC   983
SIVA          AAGGATGTTTTCNTTAATCAAGAACGAAAGTTGGGGGCTCGAAGACGATTANATACCGTCCTANTCTCAACCNTAAACGATGC   983
              .......910.......920.......930.......940.......950.......960.......970.......980...
```

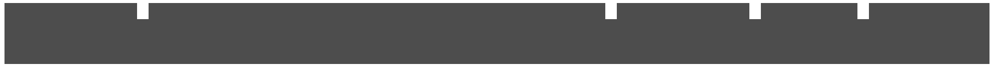

Supplement: Additional file 1 — NCBI basic local alignment search tool comparison of the S. bacillaris strain siva2011 18S rDNA sequences with different Stichococcus species. A large difference was found in the Clustal X2.0.12 multiple sequence alignment between the S. bacillaris strain siva2011 and the existing strains at nucleotides 610 to 980. SIVA, S. bacillaris strain siva2011. [file 1754-6834-7-62-S1.pdf]
